# Increasing Exploitation Durability of Two-Layer Cast Mill Rolls and Assessment of the Applicability of the XGBoost Machine Learning Method to Manage Their Quality

**DOI:** 10.3390/ma17133231

**Published:** 2024-07-01

**Authors:** Tetiana Vlasenko, Szymon Glowacki, Vitaliy Vlasovets, Taras Hutsol, Tomasz Nurek, Viktoriia Lyktei, Vasily Efremenko, Yuliya Khrunyk

**Affiliations:** 1 Department of Management, Business and Administration, State Biotechnology University, Alchevsky St., 44, 61002 Kharkiv, Ukraine; vlasenko@btu.kharkov.ua; 2 Department of Fundamentals of Engineering and Power Engineering, Institute of Mechanical Engineering, Warsaw University of Life Sciences (SGGW), 02-787 Warsaw, Poland; 3 Ukrainian University in Europe—Foundation, Balicka 116, 30-149 Krakow, Poland; 4 Department of Mechanical Engineering, Lviv National Environmental University, V. Valyki Street, 1, 80381 Dubliany, Ukraine; vlasovez@ukr.net; 5 Department of Mechanics and Agroecosystems Engineering, Polissia National University, Stary Boulevard 7, 10008 Zhytomyr, Ukraine; 6 Department of Machine Use in Agriculture, Dmytro Motornyi Tavria State Agrotechnological University, 6 Zhukovskyi Str., 66, 69002 Zaporizhzhia, Ukraine; 7 Department of Biosystem Engineering, Institute of Mechanical Engineering, Warsaw University of Life Sciences (SGGW), 02-787 Warsaw, Poland; tomasz_nurek@sggw.edu.pl; 8 Department of Power Systems Engineering, National University of Life and Environmental Sciences of Ukraine, 03041 Kyiv, Ukraine; viktoria.lyktej@nubip.edu.ua; 9 Physics Department, Pryazovskyi State Technical University, 49044 Dnipro, Ukraine; vgefremenko@gmail.com; 10 Institute of Materials Research, Slovak Academy of Sciences, 04001 Kosice, Slovakia; 11 Institute of Biochemistry, Faculty of Life Sciences, Leipzig University, Brüderstr. 34, D-04103 Leipzig, Germany; juliakhrunyk@yahoo.co.uk

**Keywords:** working layer, high-chromium cast irons, cast mill roll, exploitation durability, XGBoost algorithm, machine learning, prediction of exploitation durability

## Abstract

The increase in exploitation durability of two-layer cast rolls with the working layer made of high chromium cast iron allows one to significantly improve the quality of rolled metal as well as to increase the economic efficiency of the manufacturing process. However, it is severely hindered due to the massiveness of castings, the impossibility of both evaluating mechanical properties along the depth of the working layer, and providing the structural uniformity of the working surface and the decrease in stresses. In our research, aiming to enhance the exploitation durability of sheet rolls, it is recommended to achieve structural uniformity by CuMg alloying, which increases the concentration of copper up to 2.78 wt.% in certain zones and, owing to the accelerated austenite decomposition at a high temperature during the cool-down of the castings, led to the reduction in excessive strength and the level of heat stresses in the castings. We propose the regimes of cyclic heat treatments which, due to the decomposition of retained austenite and the fragmentation of structure, control the level of hardness to reduce and uniformize the level of stresses along the length of a barrel. A further improvement in the predictions of exploitation durability using XGboost method, which was performed based on the chemical composition of the working layer of high-chromium cast iron and heat treatment parameters, requires taking into account the factors characterizing exploitation conditions of specific rolling mills and the transformations of structural-phase state of the surface obtained by a non-destructive control method. As the controlled parameter, the hardness measured on the roll’s surface is recommended, while the gradient change in mechanical properties along the working layer depth can be feasibly analyzed by a magnetic method of coercive force measuring.

## 1. Introduction

Increased requirements with respect to the quality of mill rolls sheet rolling (rolls, in short) refer to the reliable functioning of sheet rolls [1]. While analyzing published works regarding the improvement in rolls’ mechanical properties, the following main research directions can be defined: the optimization of a material’s chemical composition and the design of new materials [2,3], the development of efficient methods of heat treatment and coating deposition [4,5], the restoration of operational conditions [4], the development of reliable methods to control the quality of working layer [5,6] of the massive cast rolls to prevent their damage in exploitation [7,8].

Conventional methods of non-destructive testing (NDT) [9], e.g., eddy current [10], radio frequency [11], heat flow thermography [12,13], optical [14], radiography [15], and acoustic emission [16] are primarily based on the search of a specific defect within the roll’s working layer. While determining the depth and extension of a localized defect, these methods, however, can be applied only to already existing defects. On the contrary, the evaluation of the structural state by a non-destructive magnet method, performed at the manufacturing stage [17], enables to choose ways optimizing the necessary mechanical properties and, hence, is more efficient. At the exploitation stage, such a method can be used to evaluate the stability of the structural state and properties, and, if necessary, based on the given data, perform regulatory impact aimed to recover operational condition, mostly by machining. 

Among the magnetic methods [18], i.e., magnetization, residual induction [19], magnetic permeability [20], Barkhausen effect [21], the method of coercive force (H_c_, A/cm) detection [22] is the most convenient for quick assessment under manufacturing conditions. This method is structure-sensitive; therefore, it can take into account a complex effect of elements during the complex alloying and heat treatment, as well as the peculiarities of the manufacturing of cast rolls having a working layer. 

The numerous works are dedicated to the advancement of the wear-resistant materials intended for the working layer of the mill rolls [23,24,25]. Such research mostly focused on the optimization of widely used materials through optimization their chemical composition and modifications, as well as the development of new steels and cast irons, including high-Cr cast irons (HCCIs) [26,27,28,29]. We studied the different mill rolls divided into four groups by their chemical composition, as follows: (i) up to 4.0 wt.% Cr; (ii) 4.0–12.0 wt.% Cr; and (iii) over 10.0–12.0 wt.% Cr. The cast irons of the first group are most frequently alloyed with nickel, molybdenum, while to a lower extent with copper, aluminum, niobium, and vanadium. They can be also modified with titanium and magnesium. The presence of graphitizing elements (Al, Ni, and Si) in their composition might lead to the graphite inclusion restricting materials’ applications [30,31]. The cast irons of the second and third groups, as a rule, do not contain graphite in the structure, they are characterized by martensite or bainite matrix with the carbide phases and retained austenite.

The coercive force methods of non-destructive testing are primarily being used for the control checking of the products. They have a limited usage in the prediction of mechanical properties and the structure of cast rolls because of the lack of corresponding systematic data on these issues. 

In [32], the measurement of coercive force was applied to reveal the significant residual stresses in large-scale cast rolls which were then released by conducting the annealing with controlled parameters. This heat treatment reduced the residual stresses by 2.6 times, which enabled the enhancement in the strength characteristics of the working layer by 20–30%. Coercive force methods are very effective to control and predict the hardness of the roll, its distribution along the length of the roll’s barrel, and the depth of the working layer. Indeed, while providing a targeted level of hardness and the uniformity of its distribution, a stable high quality of rolled steel surface is achieved. In case of deviations from uniform distribution, an uneven wear-out of the roll’s surface and variability in the thickness at the rolled sheets are observed [33]. This is mainly caused by the factors taking place during the crystallization of massive castings, which are referred to below [34]: 

The first factor: chemical segregation leading to the non-uniform concentration of alloying elements and inoculants; a possible aggregation of carbides or non-metallic inclusions. Segregation results in a structural non-uniformity and can be detected by the coercive force methods. 

The second factor: non-uniformity in heat dissipation at the crystallization which significantly affects the structure formation in the working layer, i.e., the fraction and distribution of carbide phase, the amount of austenite, etc. The increase in the volume fraction of carbide phase in local zones leads to a decrease in the exploitation durability due to the brittleness and spalling-off these zones under high load and temperature cycling. 

The third factor: a high level of local stresses mainly resulting from the deviations in heat treatments regimes during large-scale castings manufacturing.

**Figure 1:**
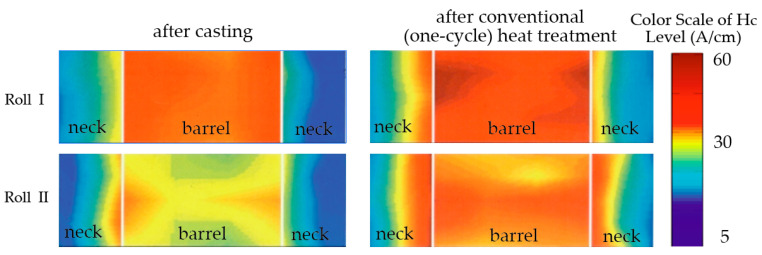
Distribution of coercive force (H_c_) across the roll’s barrel and necks [32].

In our previous research [27,32,35], we established the possibility of reliable usage of coercive force methods to reveal the uniformity of distribution of structure and hardness in the working layer of massive cast mill rolls produced by stationary or centrifugal casting. By applying the coercive force non-destructive method it was found that conventional one-step heat treatment cannot ensure the necessary level of mechanical and exploitation properties because of increasing the non-uniformity. Indeed, the investigation of the efficiency of conventional heat treatment of double layer sheet rolls casted by stationary or centrifugal casting which was conducted without taking into account tension-deformation state, led us to conclude that under such conditions, the necessary level of mechanical and exploitation properties cannot be achieved (Figure 1). In this case, a conventional one-stage treatment resulted in the increase in the properties’ non-uniformity and higher by 20% residual stresses.

In [27], the cyclic heat treatment was applied to the rolls made of high-chromium cast iron (HCCI) to increase and stabilize hardness of the working layer while reducing the residual stresses in the casting. Under the cycling, magnetostriction effect and difference in the coefficients of linear expansion of carbide and austenite were used to increase micro-stresses and to create a cellular microstructure.

The use of machine learning methods in order to predict the mechanical properties of materials is gaining increasing attention, provided that the requirements for the products’ exploitation durability during all the stages of their life cycle are stricter [36,37,38]. In particular, it is very important to predict the exploitation durability of massive cast two-layers mill rolls (with a weight of up to 18 tons), which is vitally depended on the complex of properties of alloyed working layer made of HCCI [39]. The experimental analysis of structure-phase composition and mechanical properties of the working layer requires the extracting the samples from the roll barrel’s edge. Such samples can differ regarding the structure and phase uniformity if compared with the inner zones of the working layer which results in a lower accuracy of exploitation durability forecast [40]. Therefore, any non-destructive approach to predict the roll’s properties is feasible. For this purpose, different algorithms of machine [41] and deep learning [42] are used. The possibility to predict the complex nonlinear processes of modern manufacturing, taking into account numerous technological parameters and exploitation conditions makes these methods even more appealing [43]. They allow lowering the number of costly experimental investigations based on the method of trial-and-error. 

The application of ensemble machine learning algorithms such as “Random Forest”, “eXtreme Gradient Boosting” and “Light Gradient Boosting Machine” to predict the crown accuracy of hot-rolled strips can provide a high accuracy forecast [37,44]. In particular, eXtreme Gradient Boosting model has allowed us to obtain a maximal determination coefficient of 0.97 demonstrating the highest forecasting efficiency [45,46]. The use of the algorithms, i.e., “Support vector machine”, “Multilayer perceptron”, and “XGBoost” allowed the authors to predict the values of strength and tensile strength of the products depending on the complex of technological parameters [36]. Importantly, “XGBoost” model proved to be the most accurate, and its accuracy could be further improved based on a larger set of data and input parameters. 

In order to predict the phenomenon of “strip crown” in hot rolling under the influence of many technological factors Wang et al. [37] considered the following machine learning algorithms: “XGBoost”, “Random Forest”, “Support Vector Machine” and “Multi-Layer Perceptron”. Based on the evaluation of these models with the usage of industrial data they came to the conclusion that “XGBoost” is characterized by the highest accuracy and efficiency and developed the recommendations for the intellectual preparation of steel manufacturing process. 

**Figure 2:**
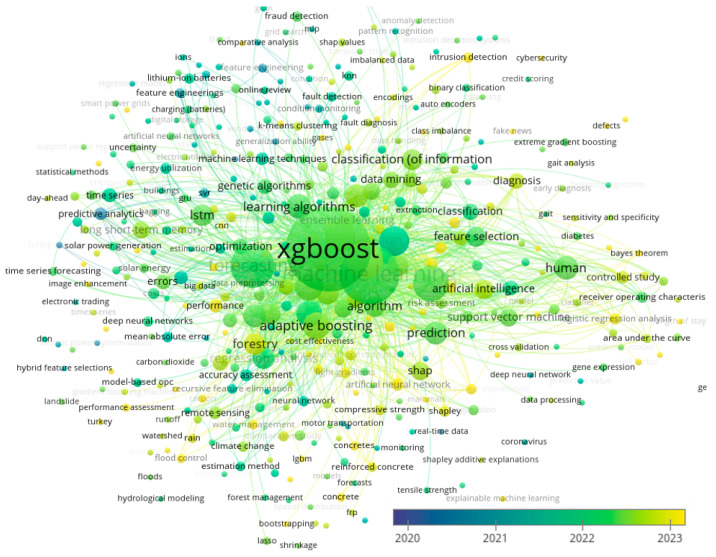
The diagram showing the clasterization of research directions in the field of machine learning and the usage of XGBoost method in the studies of materials’ properties during the period 2020–2024.

The technologies of industrial data analysis combined with the methods of machine learning represent an efficient approach [47,48,49], to determine the directions of a further increase in exploitation durability of products, while the usage of “XGBoost” model allowing, in the most cases [50], to obtain a high accuracy and the efficiency of decisions and, hence, has been intensively developing in recent years (Figure 2). Thus, the investigations of the possibility to predict the exploitation durability of sheet rolls with the working layer composed of HCCI by means of “XGBoost” machine learning model, as well as the development of the complex of approaches aimed to increase the products’ exploitation durability, has been gaining increasing interest.

An analysis of literary sources showed that there are still many reserves in matters of increasing the durability of cast mill rolls and predicting their quality and operational performance. In this regard, this work was aimed at studying approaches for improving the structure and properties of two-layer cast rolls with an HCCI working layer both through alloying and using cyclic heat treatment. Moreover, special attention was paid to the use of machine learning methods to manage the quality of rolls and predict their operational durability.

## 2. Materials and Methods

While aiming to increase the accuracy of the model based on the input data, preliminary data analysis is one of the most important steps. Hence, the input dataset on the exploitation durability of the mill rolls, which was analyzed prior to their input into the tree-based algorithms of the ensemble learning. In order to predict the exploitation durability of the rolls, the sets of real data on the exploitation durability of 382 rolls were used, following by the development of machine learning model (Table 1).

Incomplete data on the rolls, which could affect the correctness of data analysis, were removed from the list as recommended. The rest of the data underwent tests on outliers based on the Pauta Criterion [51] and Grubbs Criterion [52]. The sets of data exceeding the criteria were also excluded from the analysis. The finalized set of data used for modelling (for 370 products) was divided into learning and test sets based on the balance sampling. The ratio of the learning and test sets accounted for 80% and 20%, respectively. To prevent the impact of units of different dimensions on the accuracy of the model while conducting modeling, the input and output data were normalized as follows [1]:

(1) $$y_{i}^{\prime }=\frac{y_{i}-y_{min}}{y_{max}-y_{min}},$$

where $y_{i}^{\prime }$, *y*_*i*_, *y*_min_, and *y*_max_ are the normalized data, original data, maximal data, and minimal data, respectively.

**Figure 3:**
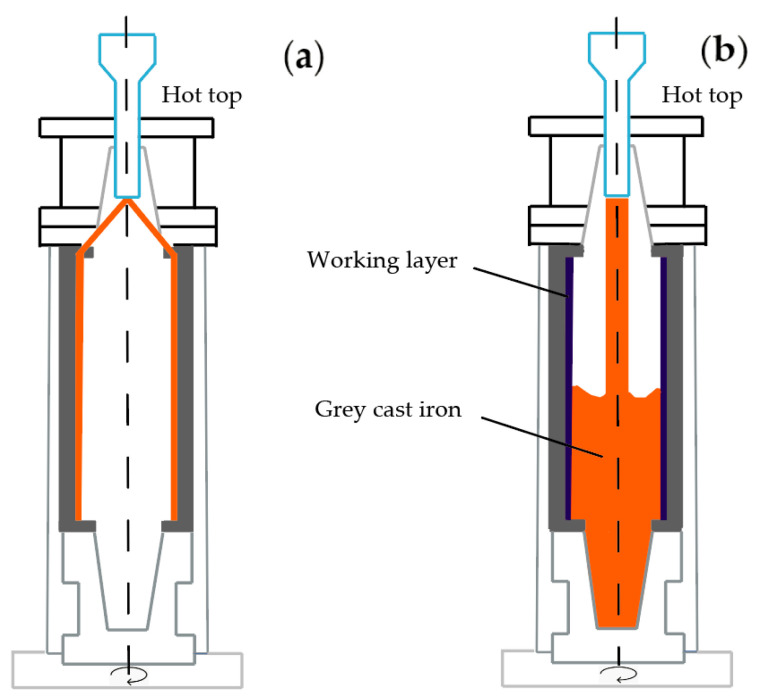
The scheme of a vertical centrifugal casting process. Casting of shell (**a**) and core (**b**) material.

**Figure 4:**
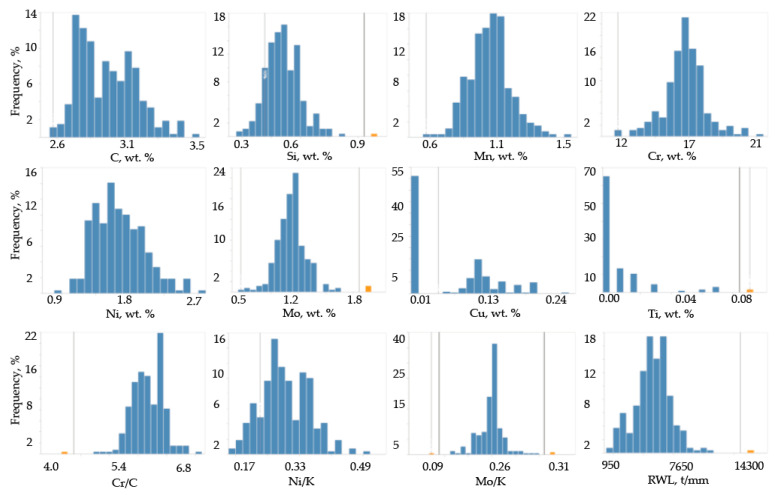
The distribution of the elements concentration in the rolls’ working layer composed of HCCI based on the analyzed dataset.

For a more accurate set of data, the rolls having a various chemical composition of the working layer have been analyzed (Table 2). The samples, collected at different stages of the manufacturing of such rolls, as well as the results obtained during the rolls’ heat treatment, were analyzed (Figure 3). A comparative analysis of rolls with various concentrations of chemical elements in the working layer was conducted (Figure 4).

The method of vertical centrifugal casting makes it possible to obtain two-layer rolls with a hard and highly wear-resistant working layer and a soft and tough core. Centrifugally cast rolls, in contrast to stationary cast rolls, have the following advantages: (a) an increased thickness of the working layer; (b) negligible gradient of hardness across the working layer due to fast solidification of a thin-walled casting; (c) a fine-grained structure due to the centrifugal forces action that prevent the formation of columnar dendrites; (d) the reduction in metal consumption by 30–40%. The use of the centrifugal casting method made it possible to select the material of the working layer and core of the barrel and roll necks regarding the specific conditions of the roll exploitation.

The smelting of metal for the working layer of two-layer cast rolls (from HCCI) was conducted in the induction furnace with the overheating up to 1530–1550 °C to reach the necessary chemical compositions. For the core of roll, grey cast iron was used; it was smelted in another induction furnace and poured into two ladles (each of 7.5 tons) at a temperature of 1460 °C. Alloying elements and fluorspar were directly added into the ladle. Then, the metal of the working layer was poured for 8–12 s into the rotating centrifugal casting machine (465–470 rpm) at the temperature of 1380–1420 °C. The used mass of HCCI metal was intended to form the rolls’ working layer of 45–50 mm thickness after finishing machining. The formation of transition zone was conducted at the lowering of centrifugation to 450 rpm and the casting of the first portion of core metal at the temperature of 1310–1320°C followed by a further holding for 4–5 min. The casting of metal (grey cast iron) from the first ladle took place at lower centrifugation, up to 350 rpm. The casting of metal (grey cast iron) of the second ladle was conducted at a gradual lowering of centrifugation up to 150 rpm (during 2.0–2.5 min), and then, in 1.0–1.5 min, at up to 50 rpm. When the centrifugal casting machine was stopped, a hot top was installed on top of the centrifugal casting machine and poured up to the height of 250–300 mm for 20–25 min. The crystallized roll was put out of casting machine and placed into the caisson where it was cooled for 96–108 h.

In order to predict the exploitation durability of sheet rolls, the data on the exploitation durability of 370 products (Table 3) was used [32].

The microstructure was analyzed using the specimens cut from the casting and directly on the surface of the rolls, using optical microscope Axiovert 40 MAT (Carl Zeiss, Iena, Germany) and the mobile microscope TKM (OM, TKM, Ukraine), as well as scanning electron microscopy (SEM) JSM-6390LV and JSM-820 (JEOL, Tokyo, Japan) equipped with an energy-dispersive X-ray detector Link AN10/85S “Link Analytical”. Analyzed surfaces were prepared by the routine procedure of polishing by the emery papers and Al_2_O_3_-containin solutions. Mirror-polished surface was etched by 4% Nital solution. The effective spot size was equal to 2–3 µm. The distribution of chemical elements was evaluated by X-ray fluorescence analyze with a X-Met 3000TXR (Oxford Instruments, Abingdon, UK) and multi-channel optical emission spectrometer (OES, SPECTROLAB F8, Kleve, Germany).

For the NDC of the structure-phase composition of the rolls’ working layer, the method of coercive force measurement (H_c_, A/cm) was applied. The measurements were conducted by employing the mobile magnetic structurescope-coercimeter (MCK, KPM, Kharkiv, Ukraine) which can measure the coercive force in the range of 1.0–60.0 A/cm and with an error not exceeding 2.5%. 

## 3. Results and Discussion

### 3.1. The Evaluation of the Capability of the XGBoost Method to Predict the Exploitation Durability of Two-Layer Cast Mill Rolls

To predict the exploitation durability of mill rolls, the “eXtreme Gradient Boost” model was used. This model is based on a set of weak models combined into a strong ensemble model by an additive method and, for the table data, it is considered to be one of the most effective modern decisions [37]. “eXtreme Gradient Boost” is parallelizing the process of a gradual tree building owing to the compatibility of cycles used for the development of basic models [1]. The external cycle determines the nodes of “leaves” of a chosen “tree”, while at another cycle (internal) the special features are tested. While the external cycle cannot be started until the internal one is not finished, the structure of cycles is regulated through the global scanning of all the inputs and the sorting through flat flows. It increases the algorithmic speed and provides a high prediction accuracy. The objective function of the method can be mathematically represented by the following equation, which includes both the loss term and the regularization term:

(2) $$Objective=\sum_{i=1}^{n}l\left(y_{i},y_{i}^{*}\right)+\sum_{i=1}^{k}\Omega \left(f_{i}\right),$$

where *n* is the number of data points; $y_{i}\;$and $y_{i}^{*}$ are measured and predicted values, respectively; *k* represents the number of trees or components in the ensemble; $\Omega \left(f_{i}\right)$ is the regularization term, penalizing complex model structures associated with individual trees.

**Figure 5:**
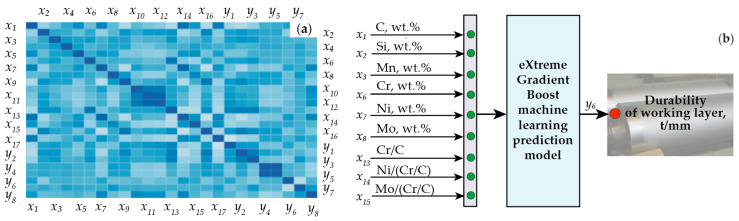
Correlation matrix of factors and indices characterizing the rolls’ exploitation durability (**a**) and input variables (x_i_) and the output parameter (y_i_) for the modeling of the exploitation durability of analyzed products by eXtreme Gradient Boost (**b**).

For the general dataset of 370 rolls (see Table 2), a correlation matrix of factors and indices characterizing the exploitation durability of the rolls were built (Figure 5).

Based on the data of correlation matrix, the finalized input data of the technological manufacturing process were selected. These input variables included the content of the following elements in the working layer (wt.%): C, Si, Mn, Cr, Ni, Mo, and the ratio of Cr/C (K = Cr/C), Ni to K coefficient (Ni/K), Mo to K coefficient (Mo/K). Selected variables are shown in Figure 4. The exploitation durability (tons/mm), being the most relevant exploitation characteristics in respect to predictability, was used as an output parameter of the model. 

**Figure 6:**
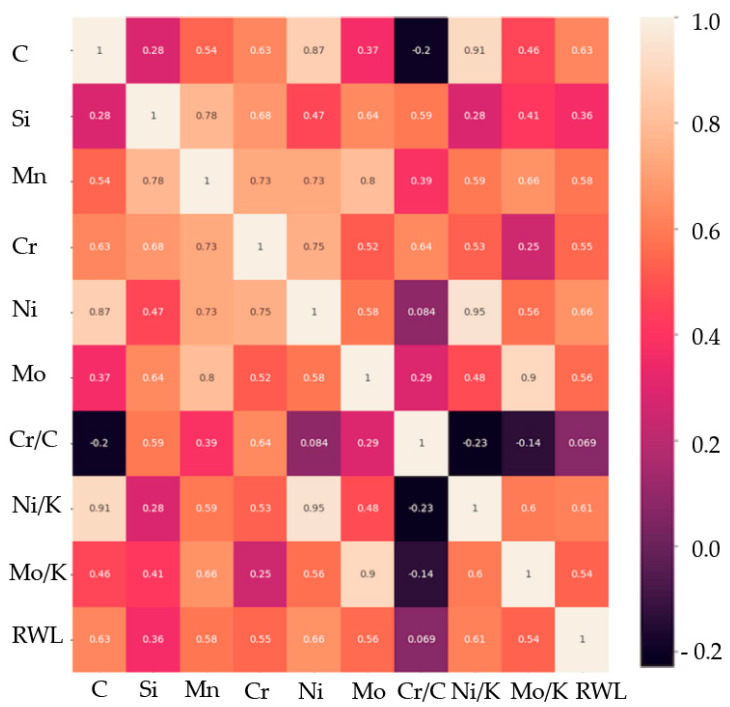
Correlation matrix of the factors and indices characterizing the exploitation durability with selected parameters for the use of eXtreme Gradient Boost regressor.

For the dataset with selected variables and the output parameter, a corrected correlation matrix was built (Figure 6). Except for Si content and the ratio of Cr to C, all the variables showed the correlation coefficient to exceed the average. 

The algorithm was implemented by PYTHON programming language (dataset parameters are shown in Table 1). To implement the algorithms for our dataset, Instance Amazon EC2 − t2 medium (compute service Amazon Web Services) which corresponded to two 3.3 GHz Intel Xeon Scalable processors (Haswell E5–2676 v3), and contained 4GB EBS memory, was suitable. 

In total, 17 input parameters (including chemical composition and heat treatment parameters) and 8 output exploitation parameters were used to predict the operational stability of sheet-rolled rolls with a working layer made of high-chromium cast iron. At the preliminary stage, the feasibility of using different models was evaluated, namely Gradient Boosting, Random Forest, Logistic Regression, eXtreme Gradient Boost. The Gradient Boosting and Random Forest models showed worse forecast accuracy compared to eXtreme Gradient Boost by an average of 10–12% with a significant increase in training time—up to 7–8 times. Although the Logistic Regression model had a shorter training time (by 16%), its accuracy was lower by 21% compared to the eXtreme Gradient Boost forecast. Therefore, it was decided to continue the research using only eXtreme Gradient Boost which had proved the highest productivity and accuracy concerning the studied conditions.

The following factors were shown to have an important impact on the accuracy of “eXtreme Gradient Boost” model prediction: the quantity of “basic estimators” (“n_estimators”), a “tree decision” depth (“max_depth”), and a “learning_rate”. For the optimization of parameters, “GridSearchCV” was employed. The finalized XGBoost parameters for the studied dataset are presented in Table 4. 

For the evaluation of model predictability efficiency using “eXtreme Gradient Boost” regressor, MSE (mean squared error), RMSE (root mean squared error), R^2^, and MAE (mean absolute error) were calculated as follows:

(3) $$MAE=\frac{\sum_{i=1}^{n}\;\left|y_{i}-y_{i}^{*}\right|}{N},$$

(4) $$MSE=\frac{\sum_{i=1}^{n}{\left(y_{i}-y_{i}^{*}\right)}^{2}}{N},$$

(5) $$R^{2}=1-\frac{\sum_{i=1}^{n}{\left(y_{i}-y_{i}^{*}\right)}^{2}}{\sum_{i=1}^{n}{\left(y_{i}-\bar{y}\right)}^{2}},$$

(6) $$RMSE=\sqrt{\frac{1}{n}\sum_{i=1}^{n}{\left(y_{i}-y_{i}^{*}\right)}^{2}},$$

where n is the number of samples; $y_{i}\;$ and $y_{i}^{*}$ are measured and predicted values, respectively.

**Figure 7:**
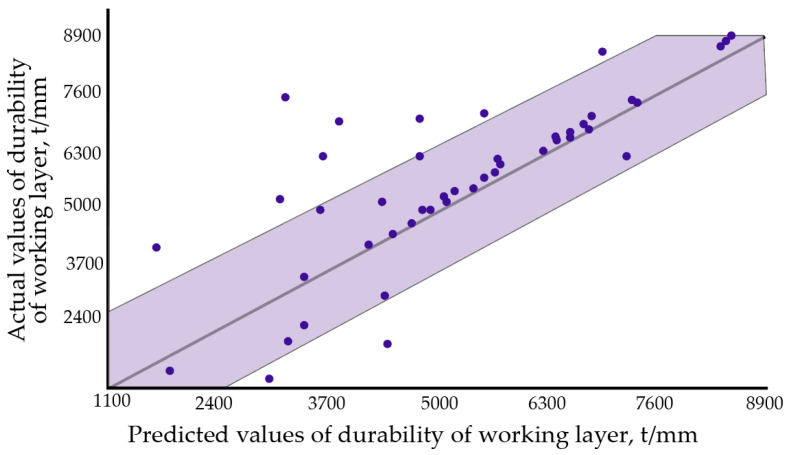
Prediction results for “eXtreme Gradient Boost”.

The results on the efficiency of “XGBoost” are presented in Table 5, while the predictability results are shown in Figure 7. 

According to Table 6 and Figure 6, the regression prediction effect of the obtained XGBoost model is sufficient to be used for the qualitative evaluation and predicting the operational stability of mill rolls having a working layer of a high-Cr cast iron. The value of the R^2^ indicator is equal to 0.61, which is appropriate for a qualitative assessment of the operational stability of the rolls. However, the accuracy of prediction may be improved if two additional indicators of structural-phase state and residual stresses are taken into account in the forecast, namely the coercive force and hardness measured on the roll’s barrel.

Usually, the hardness of the working layer of rolls is measured by the Shore method. The hardness is controlled on the surface of the roll’s barrel or on the samples extracted from the upper and lower ends of the barrel (to assess the hardness gradient along the depth of the working layer). Such samples do not reflect the hardness gradient within the working layer, liquation phenomena and structural inhomogeneities in the near-surface layer, which significantly affect the roll’s exploitation stability. Using the magnetic parameter is more expedient since it averages the value of the coercive force, both over an area of 250 mm^2^ and up to the full depth of the working layer (25–30 mm). In this case, the coercive force acts as an integral parameter describing the structural-phase composition of the material and the level of the stress–strain state in a working layer.

Using other ML models along with eXtreme Gradient Boost is feasible when processing a new set of data where these two parameters are additionally taken into account. The influence of the structural-phase state on the values of magnetic parameter and hardness will be considered in detail below.

### 3.2. The Analysis of the Structure and Phase Composition of HCCI Depending on Chromium Content

In recent years, due to the usage of centrifugal casting, highly alloyed HCCIs have been increasingly used as the working layer of two-layer rolls of the finishing broadstrip mills [27]. The limited usage of this material is connected with the low manufacturability of these alloys. One is the issue of the formation of a higher amount of retained austenite (RA) in the matrix of the HCCIs. The volume fraction of RA is caused by the concentration of C, Cr, Ni, and Mo in the alloy. In tested HCCIs, the content of C varied within the range of 2.56–3.56 wt.%. In particular, the highest number of manufactured rolls was characterized by the C concentration up to 2.75 wt.%. Cr and Ni not only increase the fraction of residual austenite, but also stabilize it, and reduce its tendency for the decomposition during the thermal treatment [53]. The content of Cr and Ni in studied HCCIs varied within the ranges of 11.8–18.8 wt.% and 0.76–2.80 wt.%, respectively. Austenite’s tendency for the decomposition can be increased by adding Mo. 

The hardness of the as-cat rolls’ working layer after the casting was within the range of 56–79 HSD, while after the heat treatment, it was within the range of 57–78 HSD. 

**Figure 8:**
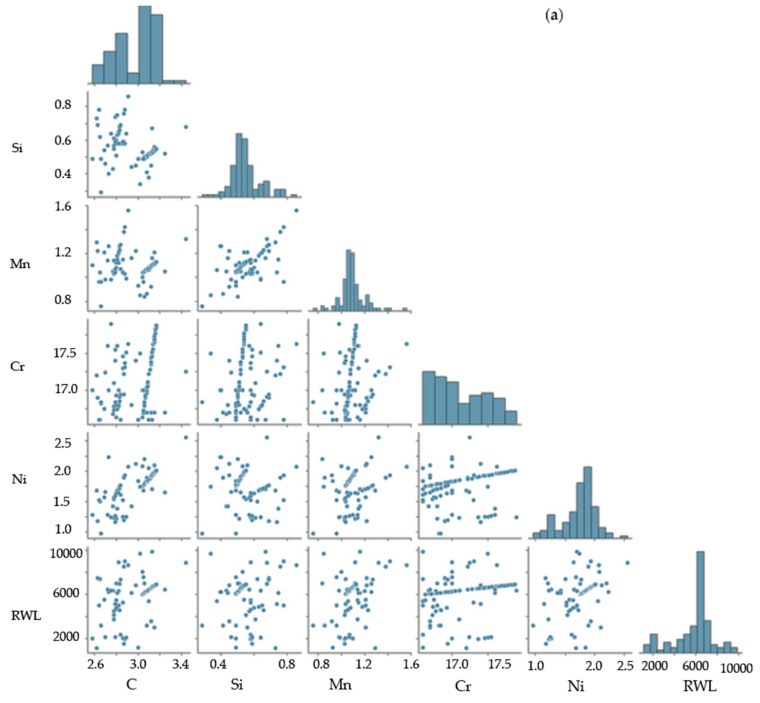

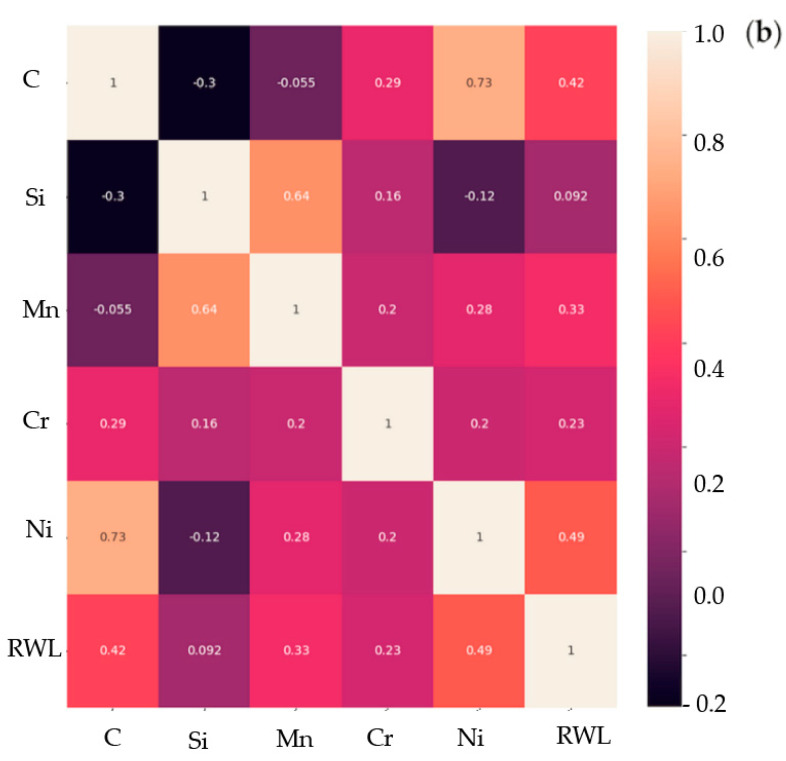
The distribution histograms (**a**) and matrices of correlation (**b**) between the studied parameters used to build machine learning models—second group (16.6–17.9 wt.% Cr).

Based on Cr content, the tested sampling was divided into three groups. For each group, the parameter distribution was studied, and the correlation matrix was built (Figure 8). The first group included the rolls containing 12–14 wt.% of Cr. The second group was composed of the rolls with 16.6–17.9 wt.% of Cr in the working layer and the concentration of other alloying elements close to the one in the first group. The third group contained the rolls with 15.9–16.4 wt.% of Cr which were additionally alloyed with Mo. The results on “XGBoost” efficiency evaluated for these groups are presented in Table 6. The evaluation of the model’s efficiency based on MSE, RMSE, R^2^, and MAE parameters indicates that dividing such cast iron into groups, solely based on the Cr concentration, is not a key decision to improving a model’s prediction, as it does not take into account the manufacturing aspects (stationary or centrifugal casting), structure and phase state, non-uniformity of structural compounds distribution, etc. 

The aspects of each group according to a conventional experimental approach using mechanical (hardness) and magnetic (coercive force) characteristics are considered below. 

**Figure 9:**
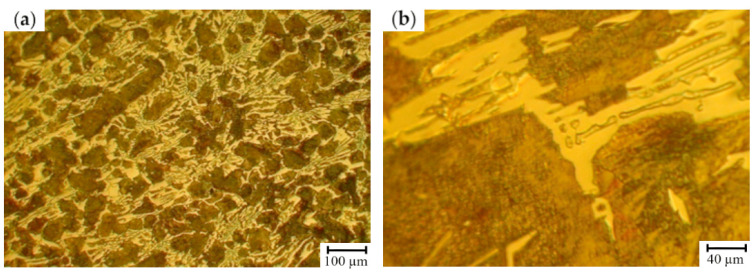
The microstructure of HCCI containing 12.0–14.0 wt.% of Cr: (**a**) total view; and (**b**) the features of carbide phase and matrix (etching by 4% Nital).

The effect of the structure on the mechanical properties of the first group of samples (12.0–14.0 wt.% Cr) was analyzed (Table 7 and Table 8). The lowest level of hardness, strength and H_c_ was typical for the cast irons which, except for troostite, cementite and austenite, are characterized by a structural constituent of dark color (after 4% Nital etching) representing the main matrix fraction which can be identified as a ferrite-carbide mixture. For the rolls with 12.0–14.0 wt.% of Cr, the retained austenite was fully decomposed (including the zones neighboring with the grains’ boundaries) during the cooling process following the casting (Figure 9). 

**Figure 10:**
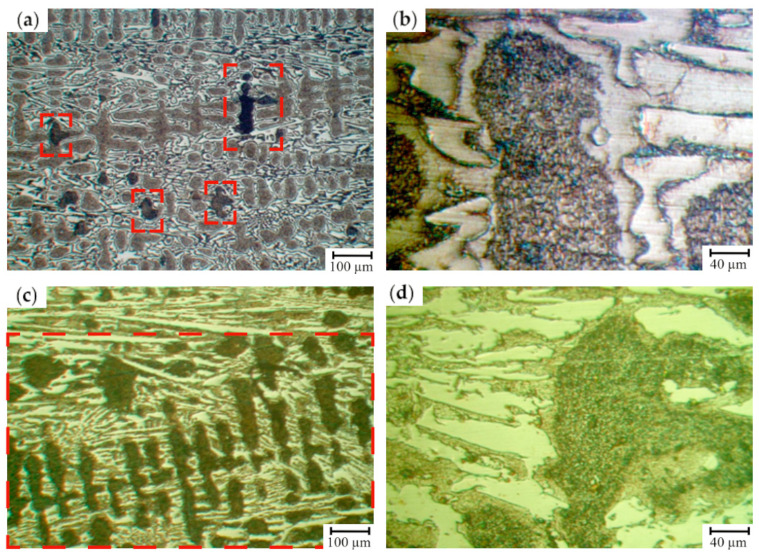
The microstructure of HCCI containing 16.6–17.9 wt.% of Cr (**a**,**b**) prior to heat treatment and (**c**,**d**) after heat treatment (annealing at 550 ± 10 °C). (**a**,**c**) are a total view, (**b**,**d**) are the features of carbide phase and matrix (etching by 4% Nital). The areas of a ferrite-carbide mixture are denoted by red dashed lines.

For the HCCIs of the second group (16.6–17.9 wt.% Cr), an essential structural non-uniformity of the matrix which is also retained after the heat treatment is typical (Figure 10). It can be assumed that the zones which are hardly etched by 4% Nital are characterized by a special structure: inside, there were the dispersed inclusions precipitated from the matrix, while at the boundaries, where the carbon concentration was lowered, austenite retained in the structure. This observation was confirmed by the non-uniform distribution of microhardness within the as-cast grain and significant increase in hardness (from 56 HSD to 78 HSD) and its uniformity after the heat treatment (annealing of 450 ± 10 °C) due to the partial decomposition of the retained austenite.

**Figure 11:**
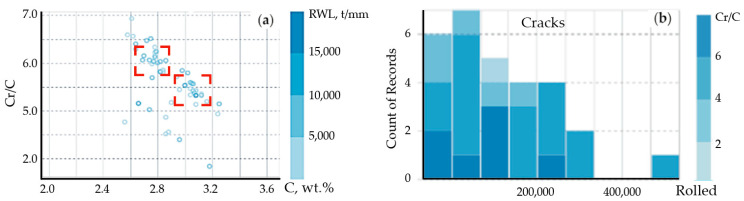
The distribution of the exploitation durability of cast mill rolls with the working layer of HCCI depending on the carbon content and the Cr/C ratio (**a**): zone I—rolls of 810 mm diameter after the one-step annealing at 450 °C; zone II—rolls of 810–920 mm diameter containing 0.10–0.19 %wt. Cu after the two-step annealing at 450 °C. The distribution of exploitation failures (superficial cracks) in cast mill rolls (**b**) with the working layer of HCCI, having different Cr/C ratio.

Figure 11 illustrates the exploitation durability of cast mill rolls with the working layer of HCCI depending on the C content and Cr/C ratio. As seen, for the rolls of 810 mm barrel diameter, the best exploitation durability values are achieved at the ratio of Cr/C = 5.2–6.3 and performing one-step annealing at 450 °C. Adding of CuMg ligatura to provide 0.1–1.9 wt.% of Cu in the working layer, ensures getting an increased exploitation durability of the rolls at a wider range of diameter, i.e., in the range of 810–920 mm. The increase in Cr/C ratio leads to the formation of a course structure, breaking off of the carbide phase and the formation of cracks on a surface.

It is known that the decomposition of retained austenite is strongly depended on chemical composition [24,54]. Thus, it is important to evaluate the concentration of alloying elements in the austenite and carbide phases. 

The EDX analysis of the HCCIs of the second group, with the step-by-step measurements across the austenite grain (each step of 5 µm), indicated that Cr concentration changes from 4.7 wt.% next to the “austenite/carbide” boundary to 14.9 wt.% Cr in the center of the grain. Manganese has a uniform distribution over the grain and its content is within the range of 0.8–1.0 wt.%. Mo and Ni were characterized by a non-uniform distribution within the matrix, i.e., the concentrations varied in the ranges of 0.6–5.2 wt.% and 0.4–2.8 wt.%, respectively. Si was fully concentrated in the matrix, in the center of the grain. In particular, in the zone, neighboring carbides, the Si fraction accounted for 1.8–2.2 wt.% while in the grain’s center it did not exceed 0.7–1.7 wt.%.

The presence of the ferrite–carbide mixture (of a dark contrast) within the structure of HCCI (was reported in cast irons of all the three groups), which was well identified on non-etched micro-sections, essentially reduced rolls’ properties including microhardness (to 110–160 HV). The presence of a ferrite-carbide mixture within the dendritic axes indicates its formation in the process of primary crystallization. With respect to a low microhardness and the presence of 5–35 vol.% of ferrite-carbide mixture in the cast iron, its impact is an essential factor affecting the properties of both as-cast and heat-treated HCCI.

**Figure 12:**
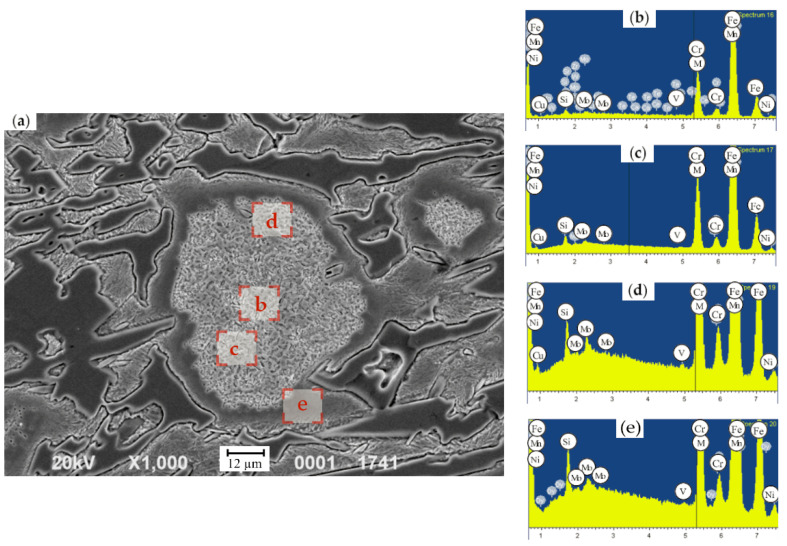
(**a**) Microstructure and (**b**,**c**) EDX spectra from different zones within the grain having a uniform austenite decomposition: (**b**)—center; c—at a distance of 20–30 µm from the center; (**d**)—at a distance of 40–50 µm from center; (**e**)—zone the structural compounds of which is hardly etched.

The EDX study of the samples extracted from the roll’s working layer containing (in wt.%) 2.77 C, 0.91 Si, 0.86 Mn, 0.064 P, 0.034 S, 17.1 Cr, 1.24 Ni, 1.17 Mo, 0.26 V, 0.33 Cu, and 0.03 Mg was conducted. In the grain’s center, where the secondary particles precipitated (Figure 12), the structure was composed of ferrite and dispersed carbides, while the concentrations of elements were the following (in wt.%): 13.11 Cr, 0.75 Mn, 1.34 Ni, 0.97 Cu. Presumably, the elevated levels of Ni and Cu can be attributed to ferrite being a part of the mixture. It is known that the solubility of Cu in α-Fe does not exceed 0.35 wt.% [55], hence more than a half of Cu (0.62 wt.% Cu) should be present in the structure in a free state. Two more EDX measurements (at a distance of 20–30 µm from the grain’s center and next to the boundary, in the zone which could not be etched) were conducted with the aim to study the uniformity of the retained austenite decomposition (Figure 11). Despite the uniform structure of this grain, essential variations in the alloying elements distribution were revealed. Thus, at a closer proximity to the boundary of the grain, the C concentration decreased almost two-fold, and Si appeared. The Si fraction in this zone accounted for 1.01 wt.%. The concentrations of Cr and Ni did not change significantly and were in the range of 12.75–13.11 wt.% (for Cr) and 1.30–1.34 wt.% (for Ni). At the grain’s boundary, the content of Cu dropped from 0.97 wt.% to 0.62 wt.%. In the center of grain, the microhardness did not exceed 207 HV; at the grain’s boundary, where the etching did not reveal the structure, retained austenite had a microhardness varying from 227–296 HV to 405–440 HV. Such an essential difference was ascribed to the variations in the content of alloying elements dissolved in the austenite. Indeed, in the zones with a high proximity to grains’ boundaries, the concentration of Cr, Mn, and Cu was about 1.5–2.0 times lower in comparison to the grain’s center, i.e., 13.11 wt.% and 7.51 wt.% (for Cr), 0.75% and 0.58 wt.% (for Mn) and 0.97 wt.% and 0.5 wt.% (for Cu). The concentration of Ni almost did not change across the grain’s cross-section varying in the limits of 1.30–1.45 wt.%.

**Figure 13:**
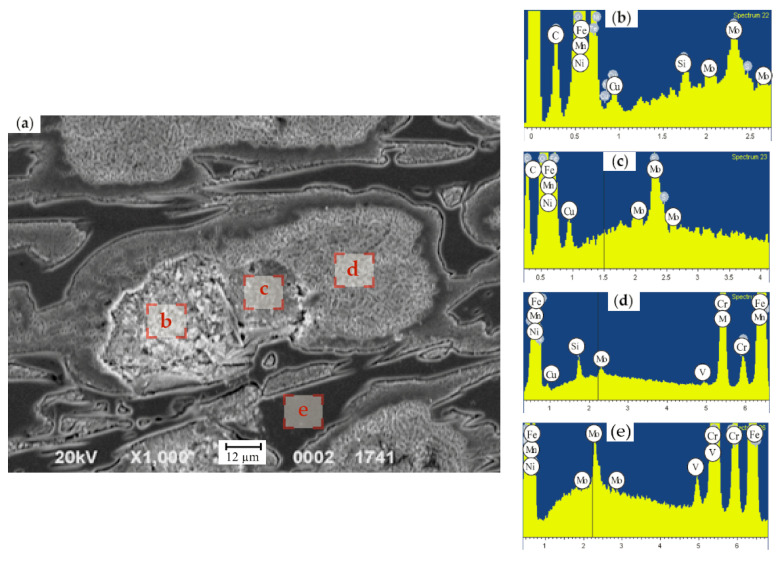
(**a**) Microstructure and (**b**,**c**) EDX spectra from different zones within the grain having a non-uniform austenite decomposition: The microstructure (**a**) and the distribution of elements (**b**–**e**) across the austenite grain with uniform dispersive hardening: (**a**) fraction of the ferrite–carbide mixture; (**b**)—fraction of the homogenous structure inside the grain; (**c**)—the fraction of dispersive hardening; and (**d**)—carbide. The etching was conducted by 4% HNO_3_.

The same EDX-study was performed for the grains having products of austenite decomposition with a non-uniform structure, as shown in Figure 13. The zones of ferrite-pearlite mixture were characterized by a dark color and the microhardness of 203–252 HV. Metallographically, this structure is obviously differentiated as the result of dispersive precipitation which occurred at a higher temperature during the cool-down, leading to a larger size of the precipitates and increased etching capacity. This zone contains (in wt.%): 28.68 Cr, 1.60 Mn, 1.27 Ni, and 1.63 Cu; the concentration of C could not be detected due to the sensitivity threshold of this method. As was shown by EDX analysis, carbon was absent in the homogenous grey areas. Hence, such inclusions could be attributed to the intermetallic phases which form under the austenite decomposition being characterized by a low microhardness. The zone of grey color contained (in wt.%) 49.73 Cr, 2.44 Mn, 2.78 Cu, and 3.81 Mo, while Ni was absent in this zone.

Taking into account the fact that, in both cases, it was the Cu concentration that increased the most, i.e., its value is 5–9 times higher in comparison to the total content of this element in HCCIs, and it can be assumed that Cu has the maximal impact on the level of austenite segregation leading to an increase in its non-uniformity during the crystallization of the working layer. 

In the grains showing the described zones, the areas of the products of the retained austenite decomposition also exist. The comparison of such zones with the grains, which are characterized by the most complete decomposition of the austenite into ferrite and dispersed carbides (Figure 13), showed an almost identical chemical composition. Such zones, being in the vicinity of homogenous grey areas, contain (in wt.%) 0.98 Si, 13.56 Cr, 0.81 Mn, 1.32 Ni, and 0.60 Cu. The concentration of the last element is two times higher than its solubility in α-Fe, which means that this zone is saturated by Cu being in a free state. 

The EDX analysis of the eutectic carbide phases allowed to identify it as a Cr-based carbide M_7_C_3_ and M_23_C_6_ with Cr and C concentrations changing within the range of 44.29–47.56 wt.% and 14.17–15.05 wt.%, respectively [56]. Also, such carbide contained 0.96–0.98 wt.% of V, 0.62–0.88 wt.% of Mn, and 1.08–2.01 wt.% of Mo. The iron content in such carbide did not exceed 36.8 wt.%. Their microhardness of the eutectic carbides varied as 797–1013 HV. In the carbide of the cementite type (observed in a minor amount), the concentration of Cr was in the range of 10–13 wt.%, which is in the agreement of the data [54]. The contents of Mn, Ni, and Mo in cementite did not exceed 0.8–1.0 wt.%. 

The initial microstructure of the working layer of the third group of HCCIs (15.9–16.4 wt.%) was represented by the carbides of different types, austenite and products of its decomposition. Here, the appearance of other stable and metastable phases (carbides, intermetallics) crystallizing due to segregation, which essentially affect the properties and the exploitation durability of mill rolls, is also possible.

### 3.3. Cyclic Heat Treatment of HCCIs

In order to select the efficient regime of heat treatment, the complex investigation of 20 regimes was completed, including one- and two-step, high- middle- and low-temperature treatments. The results on the cyclic treatment (cyclic tempering) held in the range of the temperature of magnetostriction effect of chromium carbides are presented in second table in Section 3.3. 

Retained austenite is the only phase of HCCIs ensuring the strengthening after heat treatment. Its decomposition into dispersive ferrite–carbide mixture contributes to the hardness change, the formation of uniform structure and the reduction in the tendency to crack formation. High coercive force values in as-cast HCCIs (43.8–61.5 A/cm) indicate an increased residual stresses. In this case, when the heat treatment of HCCI in the range of 450–500 °C does not lead to the change in hardness, but only reduces the stresses level, a relatively high amount of RA can be preserved in the alloy structure. It is known [57] that cyclic heat treatments more significantly change the structure and the properties of alloys while causing substructure strengthening and the elevation of strength and hardness. In HCCI, the RA is rather stable, and it can be completely decomposed only in the temperature range above 600 °C. The hardness of the working layer decreases significantly, while an intense plastic deformation and the graphitization in the roll core occur. Hence, a cyclic treatment aimed to destabilize oversaturated RA at 450–550 °C was used. 

Cycling in the range of 450–550 °C contributes to the generation of block-like structure which leads to a certain increase in hardness in comparison to one-step heat treatments [58,59,60]. Such a two-step heat treatment has the advantage of enabling the use of low-temperature equipment and to decrease the time used for heat treatment. 

In order to analyze the influence of cyclic heat treatment on the rolls’ properties, four-cycle treatment at 450 ± 10 °C was carried out (Table 9). At each cycle, the treatment lasted for 3 h. After each stage, the hardness and H_c_ were evaluated. The analyzed rolls (900 mm in diameter, 2000 mm in length) had a similar chemical composition of their working layer (wt.%): 2.70–2.77 C, 0.91–0.98 Si, 0.82–0.86 Mn, 16.3–17.1 Cr, 1.24–1.49 Ni, 1.17–1.24 Mo, 0.24–0.38 Cu, 0.23–0.31 V, and 0.03–0.04 Mg. During their casting, the thickness of the protective coating on the mold was in the range of 4.1–5.2 mm. The mold’s temperature prior to the casting was 149–166 °C. The temperature of the metal’s working layer during the casting was the same, i.e., 1420 °C. The modification of the working layer metal was conducted in a ladle by CuMg ligatura.

It was revealed (Table 9) that insignificant variations in hardness and H_c_ in both the as-cast and heat treated states are not connected with the changes in technological parameters, but are determined by the level of micro-stresses. In analyzed rolls with a similar chemical composition, the cyclic treatment not only increased the hardness, but also stabilized it, starting from the second treatment cycle (a maximum rate of dispersive precipitation is reached). A further increase in the cycle number is unfeasible as the hardness can gradually decrease. The achieved level of hardness, i.e., 84–90 HSD corresponds the coercive force of 20.1–21.0 A/cm. The analysis of samples extracted from the as-cast rolls, showed that the maximal values of H_c_ did not exceed 32.5 A/cm, while the values measured along the working layer of castings reached 43.8–61.5 A/cm. Such a difference in values could be explained by the residual stresses. In the tested rolls, the content of non-magnetic phase accounted for 11–37% (the average value is 28.4% in this group). 

The strengthening caused by the cyclic treatment can be explained by the following. Carbides being formed during the crystallization of HCCI, except for the carbide-forming elements, contain a significant amount of Fe, up to 56 wt.%. It is known that the increase in Fe in M_7_C_3_ and M_3_C carbides leads to the elevation of their magnetization [58,61,62], thus contributing into the magnetostriction effect at 450–550 °C.

Due to the changes in the coefficients of the linear expansion of the carbide phase and austenite and due to the magnetostriction effect, a cyclic treatment contributes to the increase in phase stresses (up to 700 MPa [58]), and the generation of block-like structure. Such a treatment of cast iron having a structurally free cementite leads to an additional increase in microhardness up to 30% [33] due to the phase work-hardening. Since the analyzed high-chromium rolls (manufactured at different time points) are characterized by a similar range of hardness and H_c_ values, they were united in one sampling, and the regression relationships were built. The working layer of the rolls was tested prior and after the cyclic treatment at 450 ± 10 °C.

In the as-cast rolls, with the increase in the RA volume fraction and coercive force (H_c_), the hardness after the cyclic treatment increases and can be evaluated analytically (R^2^ = 0.96) as follows:

(7) $${HSD}_{as-cast}=70+0.1\left(H_{c}\right),$$

(8) $${HSD}_{as-cast}=68+0.2\left(RA\right).$$

The high values of H_c_ in as-cast state indicates a significant level of stresses which is typical for the cast irons with a small fraction of ferrite–pearlite in the structure. Such cast irons contain a high amount of RA in the as-cast state.

The cyclic treatment leads to the increase in interphase stresses [63,64], the development of block structure [65,66], and a further decomposition of retained austenite with the precipitation of dispersed carbide phase and structure fragmentation (microhardness raises to 20–30%). In parallel to the increase in H_c_ after a cyclic treatment, a fraction of retained austenite decreases as follows:

(9) $$H_{c\;as-cast}=38.4+0.4\left(RA\right).$$

Hence, H_c_ largely depends on the fraction of retained austenite both in as-cast state and after the cyclic treatment. 

With the increased concentration of Cu (0.35–0.38%), when a large amount of RA decomposes during the cool-down of castings, the increase in annealing cycles (N) at 450 ± 10 °C leads only to the drop in hardness. In this case, hardness can be determined using the coercive force as follows:

(10) $${HSD}_{cycling}=107-0.9\left(H_{c\;as-cast}\right)-0.2(N).$$

**Figure 14:**
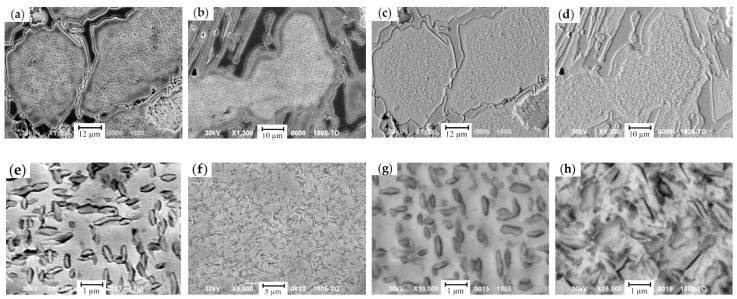
The microstructure of austenite decomposition products: (**a**–**d**) a general view; (**e**–**h**) in the grain’s center; (**a**,**c**,**e**,**g**) as-cast state; (**b**,**d**,**f**,**h**) after the cyclic treatment; (**a**,**b**,**e**,**f**—images in in secondary electrons; (**c**,**d**,**g**,**h**—images in backscattered electrons).

The effect of the cyclic heat treatment on carbide phase, austenite and its decomposition products were studied by metallographic analysis and EDX analysis (for the analysis, the samples from the top and the bottom of a roll’s barrel were selected). In the grain, which underwent a dispersive hardening following the four-cyclic treatment (Figure 14), ferrite and dispersive carbides were detected. The concentration of C in carbide phase was twice higher in comparison with the one in an as-cast state (13.11 wt.% of Cr, 1.08 wt.% of Ni) due to the increase in the carbide fraction and size. Cu and Mn were absent. The hardness increased from 311 HV to 374 HV, accounting for a 20% increase.

To determine the level of uniformity of the retained austenite decomposition, the EDX measurements in the grains with the same structure, at a distance of 20–30 µm from center and at the grain boundary, in zone which did not undergo a decomposition, were performed. As for as-cast state, essential variations in elements distribution were detected. Indeed, in the area closer to the grain’s boundary C concentration decreased by 36%. The concentration of elements up to the grain’s boundary changes as follows: the concentration of Cr dropped from 11.28 wt.% to 8.74 wt.%, concentration of Ni raised from 0.89 wt.% to 1.40 wt.%, and concentration of Mn was elevated up to 0.85 wt.%. At the grain’s boundary, the content of Cu increased up to 0.51 wt.%, while in the center, it was almost absent.

Such a change in a Cr concentration points to the possibility of the formation of carbides solely of cementite type (M_3_C), as in Cr-based carbides. The concentration of Cr reaches 43–45 wt.%. Simultaneously, the ferrite zones are saturated with Cu and its solubility limit in this phase raises up to 0.5 wt.% due to the presence of up to 1.6 wt.% of Ni. 

Close to the grain’s boundary, where the etching does not detect the structure, a retained austenite is preserved with low C and Cr concentrations which are not sufficient for decomposition (Figure 14). The hardness increase in this zone accounted for 30% (i.e., from 354 HV to 463 HV). Light areas representing a ferrite–carbide mixture, being well identified in secondary electrons, point to the non-uniformity of alloying elements distribution within the grain. 

**Figure 15:**
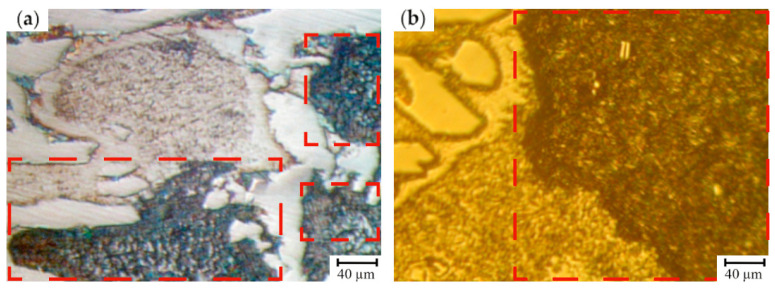
The microstructure of ferrite + carbide mixture in HCCI: (**a**) prior to the heat treatment; (**b**) after the four-cycle heat treatment. The areas of a ferrite–carbide mixture are denoted by red dashed lines.

**Figure 16:**
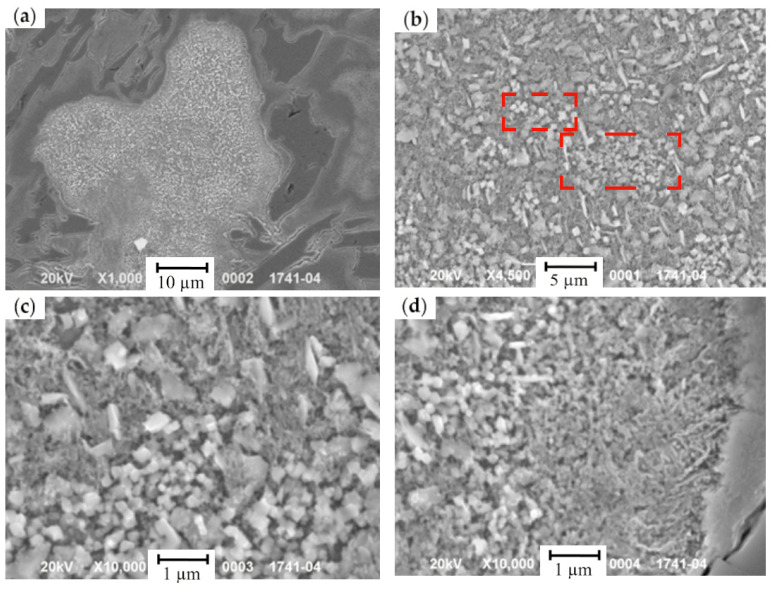
The microstructure of the areas with a ferrite–carbide mixture in secondary electrons: (**a**) a general view of the grain with a structural non-uniformity, formed during the crystallization; (**b**) carbides M_7_C_3_ in the grain’s center; (**c**) cementite carbides closer to the grain boundary; (**d**) the grain’s boundary. (The fragmentation areas are shown by red dashed lines).

During the crystallization and a further cool-down of the HCCI working layer, the zones of ferrite-carbide mixture with a reduced microhardness (108–248 HV) are being formed in dendritic axes (Figure 15 and Figure 16). The general amount of ferrite–carbide mixture after the heat treatment (in contrast to the chemical composition) did not change and accounted for 5–20%. 

**Figure 17:**
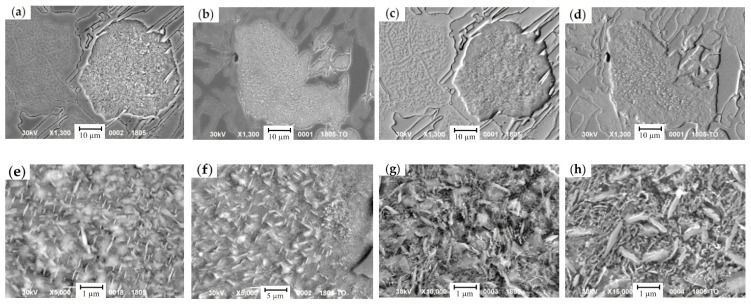
The microstructure of the areas of ferrite–carbide mixture: (**a**–**d**) a general view; (**e**–**h**) in the grain’s center; (**a**,**c**,**e**,**g**) as-cast state; (**b**,**d**,**f**,**h**) after the cyclic treatment; (**a**,**b**,**e**,**f**—images in secondary electrons; (**c**,**d**,**g**,**h**—images in backscattered electrons).

A four-cycle heat treatment contributes to the C diffusion towards the zones with increased Cu concentration. In these zones, the increase in C concentration and the enlargement of carbide precipitates (Figure 17) were detected, while ferrite was saturated by Cu (up to 0.64 wt.% of Cu). With the increase in cyclic treatment, the hardness in such zones drops to 10% (from 248 HV to 224 HV). Carbides, formed during the cyclic treatment, are finer than those appearing during the crystallization, since the latter ones were formed at a lower concentration of C and Cr. The cyclic treatment contributed to the structure fragmentation (Figure 16), and the fragments were oriented against each other at the angle of several decades of degrees. The coagulation of carbides, in comparison with the as-cast state, provided the increase in hardness and was accompanied by the decrease in concentrations of the following elements: Cr—to 37.3 wt.%, Mn—to 26.9 wt.%, and Cu—to 52.8 wt.%. Simultaneously, the concentration of Si, Ni, and Mo raised to 0.96 wt.%, 4.0 wt.%, and 1.63 wt.%, respectively.

The evaluation of the uniformity of compounds’ distribution across the grain showed that the liquation of Cr and Mo was almost absent. Simultaneously, the concentration of Si, V, and Mn decreased towards the grain’s boundary. Presumably, a joint effect of Cu, Cr and C played a significant role in the crystallization of these zones. Indeed, the Cu concentration within the grain body accounted for 0.77–0.99 wt.%, while Cu was not detected in the zone which did not undergo decomposition. However, lower concentrations of Cr (8 wt.% which is by 55% lower in comparison to the grain’s center) and of C (by 22.7%) were observed in the zone that did not decompose. 

During the crystallization of HCCI, the graphite was hardly formed, which, along with a lower heat conductivity of the material, contributes to the formation of a high level of heat stresses at the cool-down period of massive castings. To decrease the stresses, it is necessary to introduce the elements providing the formation of specific damping phases (zones) leading to the relaxation of stresses during the casting. Considering that austenite can be segregated in a liquid state due to the introduction of Cu, an experiment with adding CuMg (2.7–3.0 kg/ton) into the HCCI was conducted.

The optimization of the stress level during the cool-down (with the speed of 5–30 °C/h) of the rolls with a barrel of 700–900 mm in diameter and 2000 mm in length and containing different Cu concentrations was completed by the method of numerical modeling. The change in the speed of rolls’ cool-down within the given range of parameters can be regulated by the change in the heat transfer intensity (by the thickness of a protective coating). The impact of mentioned factors on the decomposition of RA was analyzed. It was taken into account that the increase in Cu concentration from 0.23 wt.% to 0.33 wt.% was directly connected with the RA stabilization. The increase in Cu concentration by 0.02 wt.% resulted in the RA volume fraction increase by 1.5–2.0 vol.%.

The input parameters for the optimization were as follows: the diameter of the roll’s barrel (D_b_), mm; Cu concentration (C_Cu_), wt.%; and the speed of cooling (υ_cool_), °C/h. The output parameter was represented by the level of maximal equivalent von Mises stresses (σ_e_), MPa. A design of planning experiment to obtain a mathematical relation σ_e_ = f (D_b_, C_Cu_, υ_cool_) was completed. To develop a mathematical model, the rotatable plan of a second order, of three factors, was selected. Factor coding was conducted to build the plan-matrix of the experiment (Table 10).

**Figure 18:**
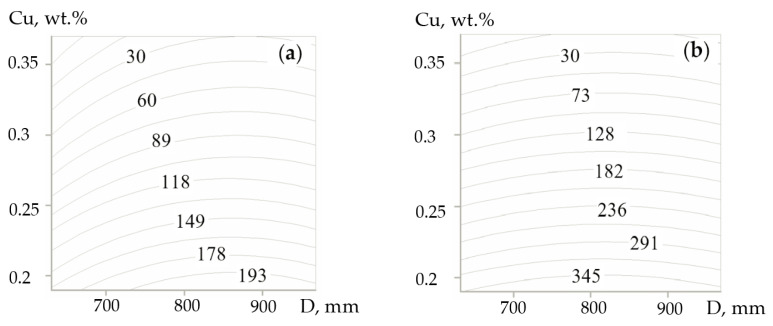
The effect of the diameter of a roll’s barrel and Cu concentration on the level of von Mises maximal equivalent stresses during the cool-down with a speed of 10 °C/h (**a**) and 40 °C/h (**b**).

**Figure 19:**
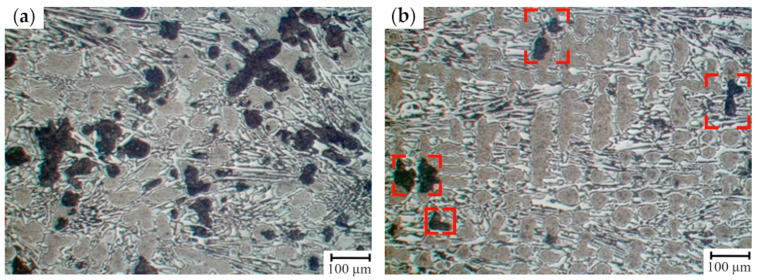
The microstructure of HCCI containing 0.25–0.28 wt.% Cu with different carbon content: (**a**) 2.84 wt.%, (**b**) 2.68 wt.%. The areas of a ferrite-carbide mixture are denoted by red dashed lines.

As follows from Figure 18, the surface of the response had a shape of the elliptic paraboloid with a long axis oriented towards the change of roll’s diameter. It illustrates an essential influence of Cu concentration on the level of stresses occurred under the post-casting cooling. With the increased C concentration (2.73–2.90 wt.%), during the cool-down of the casting and Cu liquation, the austenite undergoes a decomposition into the ferrite-carbide mixture of a dark contrast (Figure 19). At the concentration of C in the range of 2.62–2.72 wt.%, such zones were in a minor amount in the structure. 

The mathematical processing of the experimental results allowed obtaining the following regression equation:

(11) $$\sigma_{e}=\;135.6+8.19X_{1}-67.87X_{2}+26.43X_{3}+0.79X_{1}X_{2}-2.41X_{1}X_{3}-18.54X_{2}X_{3}-7.70X_{12}+3.46X_{22}-3.98X_{32}$$

Therefore, the introduction of CuMg alloying increases the local concentration of Cu and enables, while regulating the austenite content, to reduce the hardness and the level of heat stresses in the castings. In order to provide a high level of HCCIs’ hardness, the concentration of C must be limited to 2.6 wt.%, or it is necessary to conduct a cyclic heat treatment, i.e., a two-step annealing at 450 ± 10 °C providing the diffusion of C and Cu, and the formation of Cr-based carbides even in liquation zones. 

The EDX analysis of eutectic carbides after the heat treatment showed that, in comparison to their as-cast state, their chemical composition was slightly changed, i.e., the concentration of Cr decreased from 46.3–47.6 wt.% to 43.1–45.6 wt.%, whereas the concentration of C rose by 1.1–1.3 wt.%. Also, such carbides contained 1.08–1.27 wt.% of V, 0.97–1.00 wt.% of Mn, and 1.49–1.69 wt.% of Mo. The fraction of Fe did not exceed 43.1 wt.%. The X-ray diffraction analysis revealed that the cyclic heat treatment at 450 ± 10 °C led to almost a complete decomposition of retained austenite (the remaining fraction did not exceed 2–4 vol.%). 

The carbide phase precipitated during the crystallization and annealing corresponded to the Cr_7_C_3_ type carbide. These carbides are characterized by a low coefficient of magnetic anisotropy, hence, even when they are present as dispersed particles in a high quantity, which is evidenced by the increase in hardness, they do not essentially affect the level of coercive force. The assumptions postulated by several researchers [2,24,54], according to which heat treatment leads to the transition of a carbide Cr_7_C_3_ into a softer one, Cr_23_C_6_, and then to Me_3_C, which would negatively affect the exploitation durability of the HCCI working layer, were not confirmed in the present research.

Based on the results of our investigations aiming to analyze the exploitation durability of cast rolls, it was shown that the rolls (815 mm in diameter, 2000 mm long) with the HCCI working layer manufactured by centrifugal casting, are characterized by lower hardness parameters, in average by 10 HSD in comparison to those which were obtained by stationary casting. However, the exploitation durability of the rolls obtained by both casting types was comparable. The centrifugally cast rolls of a larger diameter (900 mm in diameter, 2000 mm long) in comparison to those obtained by stationary casting, at the hardness of 63 HSD and 73 HSD, respectively, showed a higher operating time, i.e., by 5.3–10.0%. This is due to the fact that the working layer of these rolls had more uniform depth. 

The highest exploitation durability was shown for the centrifugally cast rolls DLCr17NiMo-63, of 900 mm diameter ×2000 mm in length (operating capacity of 190,623 ton/roll), and for stationary-cast rolls DLCrNi-63 (operating capacity of 180,932 ton/roll). After the completion of exploitation observations, all the centrifugal-casted rolls DLCr17NiMo-63 had a working layer of depth sufficient for the restoration by turning, while only 65% of the stationary-cast DLCrNi-63 rolls demonstrated the possibility of being restored. The controlled rolls also showed minor breakages (1–2 rolls), spalling-off (1–2 rolls) and cracks (2–3 rolls), though the number of rolls showing net shaped microcracks accounted for 9–11 rolls (8–9%). More coarse carbides led to the breaking off at the working surface in 7 rolls (9–10%) of stationary-cast DLCrNi-63 rolls. 

Centrifugally cast rolls DLCr17NiMo-58 were less prone to be damaged (breaking off, cracks, spalling-off, the formation of net shaped microcracks of the working layer), i.e., only 1 roll per each type of damage was recorded. The increase in rolls’ hardness (up to 73 HSD) independently from the type of their manufacturing (centrifugal vs. stationary casting) increases the incidence of spalling-off (8 and 35 rolls, respectively), breaking off (5 and 10 rolls, respectively), the formation of net-shaped microcracks on the roll’s surface (2 and 24 rolls, respectively). In comparison to the stationary-cast rolls, the centrifugally cast ones, independent of their type and size, in most cases lasted until the full wear-out of the working layer. 

In future investigations, the introduction to the prediction models of such characteristics as surface hardness and the integral parameter of structure-phase state, which is determined by a coercive force (takes into account the gradient of properties at the depth of the working layer), will allow one to develop the strategies to optimize chemical composition, manufacturing parameters and define the optimal interval parameters with the objective of increasing the exploitation durability of mill rolls and improving the quality of the rolled metal. 

## 4. Conclusions

The application of two-layer cast rolls with a working layer made of high-Cr cast iron increases their exploitation durability by about 1.5 times compared to forged steel rolls. This is due to uniformity of structure across the working layer, which can be evaluated by a non-destructive method of the coercive force measurement. This method allows the optimization of the heat treatment parameters to ensure a specific ratio of microstructural constituents. 

In the as-cast HCCI rolls’ working layer, hardness varies in the range of 62–79 HSD. The hardness can be improved by the regulation of the retained austenite volume fraction. Using the measurement gives the most reliable information on RA since the coercive force is very sensitive to the structural components variation.

Copper in amounts of 0.21–1.17 wt.%, added to HHCI cast rolls to modify its structure, is the most important factor contributing to the non-uniform formation of a roll’s structure. This is attributed to its lower solubility in α-Fe and carbide phases, thus promoting the formation of segregation zones in RA where carbon is hardly present. The addition of the CuMg ligature increases the local concentration of Cu in austenite up to 2.78 wt.%, which accelerates the austenite decomposition under the cool-down of castings (at 750–600 °C). This decreases the hardness and residual stresses in the cast roll.

The cycling heat treatment with annealing at 450–550 °C leads to an additional increase in the hardness of the working layer due to the more complete decomposition of austenite induced by the magnetostriction effect.

When predicting the durability of two-layer cast mill rolls with a high-Cr working layer, the “eXtreme Gradient Boost” ensemble model performed the average level of prediction of R^2^ = 0.61 which is considered acceptable to be used in the manufacturing processes. The features of a specific rolling mill exploitation, as well as the structure of the working layer should be additionally considered to further improve the model.

Hardness, structural gradient, and residual stresses in the working layer of the mill rolls are advised to be controlled by the non-destructive method based on the measurement of the integral magnetic parameter (coercive force).

## Figures and Tables

**Table 1:** The list of inputs and outputs of the prediction model for the exploitation durability of sheet rolls with a working layer of HCCI ^1^.

Values	Features	Unit	Designations	Distribution Limits	Symbol
Manufacturing stage	C	wt.%	*x*_1_	2.56–3.56	C
	Si	wt.%	*x*_2_	0.24–0.98	Si
	Mn	wt.%	*x*_3_	0.59–1.56	Mn
	P	wt.%	*x*_4_	0.03–0.23	P
	S	wt.%	*x*_5_	0.01–0.06	S
	Cr	wt.%	*x*_6_	11.8–18.8	Cr
	Ni	wt.%	*x*_7_	0.76–2.8	Ni
	Mo	wt.%	*x*_8_	0.47–1.44	Mo
	Mg	wt.%	*x*_9_	0.0–0.04	Mg
	Cu	wt.%	*x*_10_	0.04–1.17	Cu
	Ti	wt.%	*x*_11_	0.006–0.11	Ti
	V	wt.%	*x*_12_	0.01–0.44	V
	Cr/C	-	*x*_13_	3.83–6.93	Cr_C
	Ni/(Cr/C)	-	*x*_14_	0.18–0.45	Ni_K
	Mo/(Cr/C)	-	*x*_15_	0.1–0.33	Mo_K
	Annealing temperature	°C	*x*_16_	450–500	annealing
	The temperature of cyclic treatment	°C	*x*_17_	400–700	double_annealing
Exploitation	Barrel diameter (start)	mm	*y*_1_	664–915	dstart
	Barrel diameter (after exploitation)	mm	*y*_2_	663–909	dfinish
	Number of roll changes	pcs	*y*_3_	1–207	number_transhipm
	Rolled	ton	*y*_4_	2190–464,255	rolled
	The thickness of removed layer per one roll change	mm	*y*_5_	0.18–3.60	removal_transshipm
	Durability of working layer	ton/mm	*y*_6_	206–17,600	RWL_tmm
	Durability of working layer	ton	*y*_7_	1260–3750	RWL_tlayer
	The state of a barrel surface after the exploitation	See footnote ^1^	*y*_8–14_	Scrap—14.5%	Condition
				Club—12.7%	
				Wear—10.9%	
				Barrel—1.82%	
				Breaking—1.82%	
				Spalling-off—1.82%	
				Neck—1.82%	

^1^ On the barrel surface of a sheet roll the following defects were detected after the exploitation: insignificant traces of wear (exploitation tests continue), fractures, shrink-holes, the damages along the roll wobbler, the damages of the roll barrel. The set of data only includes the rolls which underwent two cycles of annealing at the same temperatures.

**Table 2:** The chemical composition of the working layer of mill rolls (wt.%).

Value	C	Si	Mn	Ni	Cr	Mo	Ti	V	Cu
The total scatter	2.56–3.56	0.24–0.98	0.59–1.56	0.76–2.8	11.8–18.8	0.47–1.44	0.006–0.11	0.01–0.44	0.04–1.17
Mean	2.75	0.84	0.93	1.37	16.50	1.11	0.01	0.26	0.22

Note: The concentrations of sulphur and phosphorous were 0.01–0.06 wt.% and 0.03–0.23 wt.%, respectively.

**Table 3:** Average rolls working life and the reasons for decommissioning.

Roll Designation	Size	Chips	Cracks	Breakdowns/ Detachments	Grid Cracks	Average Operating Time on the Roll	Total Rolls Analyzed
Centrifugal Cast							
DLCrNiMo—73 ^1^	820 × 2000	5	2	–/8	2	153,800	37
DLCr17NiMo—58 ^2^	900 × 2000	1	1	–/2	1	139,738	14
DLCr17NiMo—63 ^2^	900 × 2000	2	3	1/2	9	190,623	38
DLCr17NiMo—63 ^2^	820 × 2300	–	1	–/–	2	140,194	6
Stationary Cast							
DLCrNi—63 ^3^	900 × 2000	7	2	–/1	11	180,932	75
DLCrNiMo—73 ^4^	820 × 2000	8	–	2/32	9	140,980	110
DLCrNiMo—73 ^4^	820 × 2300	2	–	1/3	15	147,335	90

Two-layer mill rolls with the core from grey iron cast: ^1^—of centrifugal casting, with Cr–Ni–Mo cast iron working layer, with the surface hardness of 73HSD; ^2^—of centrifugal casting, with the working layer of HCCI, surface hardness of 58HSD and 63HSD, respectively; ^3^—of stationary casting with the working layer of Cr–Ni cast iron, surface hardness of 63 HSD; ^4^—of stationary casting with the working layer of Cr–Ni–Mo cast iron, surface hardness of 73HSD.

**Table 4:** The parameters of the “eXtreme Gradient Boost” model.

Parameter	Value
n_estimators	80
max_depth	50
learning_rate	0.1

**Table 5:** The results on the efficiency of “XGBoost” model.

Parameter	Value
MSE (mean squared error)	1,196,491
RMSE (root mean squared error)	1094
R^2^	0.61
MAE (mean absolute error)	617

**Table 6:** The results on the “XGBoost” model efficiency evaluated for HCCIs divided based on the chromium concentration.

Parameters	Sample Group		
	12.0–14.0 wt.% Cr	16.6–17.9 wt.% Cr	15.9–16.4 wt.% Cr with Mo
MSE	1,828,335	690,033	1,579,079
RMSE	1352	830	1256
R^2^	0.344	0.396	0.254
MAE	740	516	933

**Table 7:** The ratio of phases in HCCI with 12.0–14.0% Cr after the casting.

Designation of Roll	The Volume Fraction of Structural Constituents, vol.%			
	Troostite	Ferrite–Carbide Mixture, Characterized by Weak Etching	Cementite	Austenite
1	33.0	35.0	18.0	14.0
2	49.0	3.5	22.5	28.5
3	52.4	7.0	18.0	22.6
4	71.2	5.5	12.0	11.3
5	57.5	6.2	20.3	16.0

**Table 8:** The properties of the as-cast HCCI rolls’ working layer containing 12.0–14.0% of Cr.

Designation of Roll	Properties			
	HSD	UTS, MPa	Ultimate Strength under Compression, MPa	Coercive Force, A/cm
1	61.0	708	2360	17.6
2	56.0	936	3120	46.4
3	65.0	780	2600	22.9
4	65.0	768	2560	22.7
5	63.0	756	2520	19.4

**Table 9:** The properties of HCCI in as-cast state and after the cyclic treatment at 450 ± 10 °C.

The Roll Designation	As-Cast State		After the Number of Cycles							
			I		II		III		IV	
	Hc, A/cm	HSD	Hc, A/cm	HSD	Hc, A/cm	HSD	Hc, A/cm	HSD	Hc, A/cm	HSD
1	21.9–22.2 22.1	75–75 75	20.2–20.4 20.4	84–84 84	20.7–20.8 20.7	89–91 90	20.3–20.4 20.4	84–84 84	20.5–20.8 20.6	86–87 87
2	24.5–24.8 24.7	81–81 81	21.4–21.8 21.6	84–84 84	20.1–20.3 20.2	85–85 85	19.5–19.8 19.6	84–84 84	19.3–19.4 19.4	81–83 82
3	25.4–25.7 25.5	75–75 75	22.2–22.3 22.3	82–84 83	20.6–21.0 20.8	84–84 84	21.0–21.0 21.0	84–84 84	20.4–20.5 20.4	81–83 82

In a numerator, minimal and maximal values are shown, and in a denominator, average characteristics are shown.

**Table 10:** Factor coding during the optimization of the level of stresses within the working layer of HCCI rolls.

Variability Interval and the Level of Factors	Diameter of a Roll’s Barrel, mm	Cu Concentration, %	Cool-Down Speed, °C/h
Zero level	800	0.28	20
Variability interval	100	0.05	10
Low level	700	0.23	10
Upper level	900	0.33	30
Reference point −1.682	630	0.19	5
Reference point +1.682	970	0.37	40
Coded value	X_1_	X_2_	X_3_

## Data Availability

The original contributions presented in the study are included in the article, further inquiries can be directed to the corresponding authors.
